# Production of isoprene, one of the high-density fuel precursors, from peanut hull using the high-efficient lignin-removal pretreatment method

**DOI:** 10.1186/s13068-017-0988-5

**Published:** 2017-12-08

**Authors:** Sumeng Wang, Zhaobao Wang, Yongchao Wang, Qingjuan Nie, Xiaohua Yi, Wei Ge, Jianming Yang, Mo Xian

**Affiliations:** 1 Shandong Key Lab of Applied Mycology, College of Life Science, Qingdao Agricultural University, No. 700 Changcheng Road, Chengyang District, Qingdao, 266109 China; 2 Foreign Languages School, Qingdao Agricultural University, Qingdao, 266109 China; 3 CAS Key Laboratory of Biobased Materials, Qingdao Institute of Bioenergy and Bioprocess Technology, Chinese Academy of Sciences, Qingdao, 266101 China

**Keywords:** Isoprene, Peanut hull, Pretreatment, Phosphoric acid/hydrogen peroxide, Lignin removal, Enzymatic hydrolysis efficiency

## Abstract

**Background:**

Isoprene as the feedstock can be used to produce renewable energy fuels, providing an alternative to replace the rapidly depleting fossil fuels. However, traditional method for isoprene production could not meet the demands for low-energy consumption and environment-friendliness. Moreover, most of the previous studies focused on biofuel production out of lignocellulosic materials such as wood, rice straw, corn cob, while few studies concentrated on biofuel production using peanut hull (PH). As is known, China is the largest peanut producer in the globe with an extremely considerable amount of PH to be produced each year. Therefore, a novel, renewable, and environment-friendly pretreatment strategy to increase the enzymatic hydrolysis efficiency of cellulose and reduce the inhibitors generation was developed to convert PH into isoprene.

**Results:**

The optimal pretreatment conditions were 100 °C, 60 min, 10% (w/v) solid loading with a 2:8 volume ratio of phosphoric acid and of hydrogen peroxide. In comparison with the raw PH, the hemicellulose and lignin were reduced to 85.0 and 98.0%, respectively. The cellulose–glucose conversion of pretreated PH reached up to 95.0% in contrast to that of the raw PH (19.1%). Only three kinds of inhibitors including formic acid, levulinic acid, and a little furfural were formed during the pretreatment process, whose concentrations were too low to inhibit the isoprene yield for *Escherichia coli* fermentation. Moreover, compared with the isoprene yield of pure glucose fermentation (298 ± 9 mg/L), 249 ± 6.7 and 294 ± 8.3 mg/L of isoprene were produced using the pretreated PH as the carbon source by the engineered strain via separate hydrolysis and fermentation and simultaneous saccharification and fermentation (SSF) methods, respectively. The isoprene production via SSF had a 9.8% glucose–isoprene conversion which was equivalent to 98.8% of isoprene production via the pure glucose fermentation.

**Conclusions:**

The optimized phosphoric acid/hydrogen peroxide combination pretreatment approach was proved effective to remove lignin and hemicellulose from lignocellulosic materials. Meanwhile, the pretreated PH could be converted into isoprene efficiently in the engineered *Escherichia coli*. It is concluded that this novel strategy of isoprene production using lignocellulosic materials pretreated by phosphoric acid/hydrogen peroxide is a promising alternative to isoprene production using traditional way which can fully utilize non-renewable fossil sources.

## Background

With the repaid development of modern industry and global economy, the demand for the fuels is always on the rise. However, a fact cannot be denied that the non-renewable resources might be exhausted in the near future at such an alarming consuming rate. Additionally, the consumption of these non-renewable resources on a large scale can also lead to severe environmental problem such as global warming. Therefore, it is necessary to produce biofuels with minimal environmental impact from renewable sources to close the “energy gap” between the global supply and demand. Apart from the minimal impact on the environment, biofuels can also eliminate greenhouse gases release [1, 2]. Based on the above, to explore a new strategy to produce renewable energy in place of the traditional non-renewable energy has become an urgent issue.

Compared with the first generation biofuels production derived from grain, the second generation biofuels produced from plant lignocellulose are more promising. The most desirable biomass energy sources are lignocellulosic materials including wood, agricultural crops, and their waste by-products [3]. They are cheaper, abundant, and renewable [4, 5]. And the high yield of annual 220 billion tons in particular undoubtedly provides a guarantee for these lignocellulosic materials to be the largest biomass source in the world currently [6]. Therefore, lignocellulosic materials are supposed to be the potential alternative to biofuels production such as bio-isoprene and ethanol once derived from fossil sources. However, few studies have focused on the biofuel production using the lignocellulosic material peanut hull (PH) as the carbon source by far [7]. As we all know, China is the largest peanut producer in the world, accounting for about 40% of global yield (32.22 million tons annually) [8], which provides possibility for biofuels production derived from PH.

Isoprene (C_5_H_8_), 2-methyl-1,3-butadiene, is the monomeric five-carbon building block for different kinds of naturally occurring compounds, which not only could serve as feedstock in synthetic chemistry industry such as rubber, but also has significant potential to produce a renewable drop-in biofuel [1, 2, 9–11]. Since isoprene has double bond and branched chain structure, it is easy to generate polymers with ring structure [12]. On the one hand, the oligomerization of isoprene units has been used to generate second-order fuel molecules which could be regarded as the supplements of gasoline, jet fuel, and diesel [9]. On the other hand, terpenes such as α-pinene, β-pinene, camphene, and limonene are the dimers of isoprene. Interestingly, these dimers mixed with a proper proportion could form high-density fuel products through a variety of acidic catalyst and hydrogenation catalyst [13].

Isoprene was first detected as a volatile substance from the leaves of acacia, poplar, oak, and other plants via the photosynthesis process [14]. Notably, it is easy for isoprene to pass through chloroplast and cellular membranes and finally get released into atmosphere due to its hydrophobic and volatile properties [9, 15]. However, since it is difficult to harvest isoprene from plant species [16], plants would not be the primary source to produce isoprene. Currently, the isoprene production mainly relies on fossil sources via chemical methods, especially the petroleum-based feedstock. However, the depletion of non-renewable fossil sources would limit its development and application in the future [9, 17]. Furthermore, isoprene production through chemical methods will also lead to severe environment pollution. Therefore, it is urgent to develop an environment-friendly, low-energy consumption, and high-production method to replace the traditional chemical methods. Here, we have developed more environmental-friendly process and technology that produce isoprene from the cheap and renewable materials than traditional strategy [18]. In the past decades, many kinds of microorganisms, such as *Bacillus subtilis*, *Saccharomyces cerevisiae*, *Escherichia coli* (*E. coli*), and microalgae, have been reported to be able to produce isoprene with different yield efficiencies [1, 19–21]. Worth mentioning, in our previous study, the current highest glucose–isoprene conversion (7%), corresponding to a 6.3 g/L concentration of isoprene, was achieved using the engineered strain YJM25 (*E. coli* BL21™ (DE3)/pYJM21, pYJM14) [21].

At present, almost all the isoprene-producing research with biological methods aimed to convert pure glucose into bio-isoprene with microorganisms. Only a few studies focus on producing bio-isoprene using lignocellulose as the carbon source [22]. The primary reason is that it is very difficult to utilize the lignocellulosic materials directly because the cellulose is surrounded by hemicellulose and lignin [23] and a huge amount of enzyme would be consumed to hydrolyze cellulose contained in the lignocellulosic materials. Thus, it is vital to remove lignin and hemicellulose from lignocellulosic materials by an appropriate pretreatment method before isoprene production with microorganism fermentation.

Up till now, many pretreatment methods like chemical (dilute acid and alkali), physical (grinding and popping), and biological methods, have been explored to improve the enzymatic hydrolysis efficiency of cellulose by means of reducing the hemicellulose and lignin of lignocellulosic materials [24–31]. It was reported that the hemicellulose and lignin of lignocellulose could be separately removed by dilute acid and hydrogen peroxide [32, 33]. Meanwhile, during the pretreatment process, there are various inhibitors formed, which could influence the following fermentation [34]. In this study, a promising pretreatment method, combination of phosphoric acid (H_3_PO_4_) and hydrogen peroxide (H_2_O_2_) (H_3_PO_4_/H_2_O_2_), was introduced to remove hemicellulose and lignin of the raw PH. In spite of the fact that the price of industrial grade H_3_PO_4_ is higher than sulfuric acid, H_3_PO_4_ is still considered to be the proper reagent for pretreatment because of its greater advantages, such as less corrosivity, less toxicity, lower environment impact, and being a source of phosphorous as a nutrient for microorganisms [35]. The pretreatment method developed in this study had greater advantages over others because it could remove more hemicellulose (85.0% w/w) and lignin (98.0% w/w), increase enzymatic hydrolysis efficiency (95.0% w/w) of cellulose, and form fewer inhibitors (1.62 mg formic acid, 6.88 mg levulinic acid, and 8.56 × 10^−4^ furfural per gram of pretreated PH, respectively). Consequently, PH pretreated by this combination method could be used to produce the high-density fuel precursor (isoprene) by the engineered *E. coli*. Aside from that, the promising pretreatment method also has bright prospects as to be applied to pretreat other lignocellulosic materials in the future.

## Results and discussion

### Optimization of pretreatment conditions

**Fig. 1:**
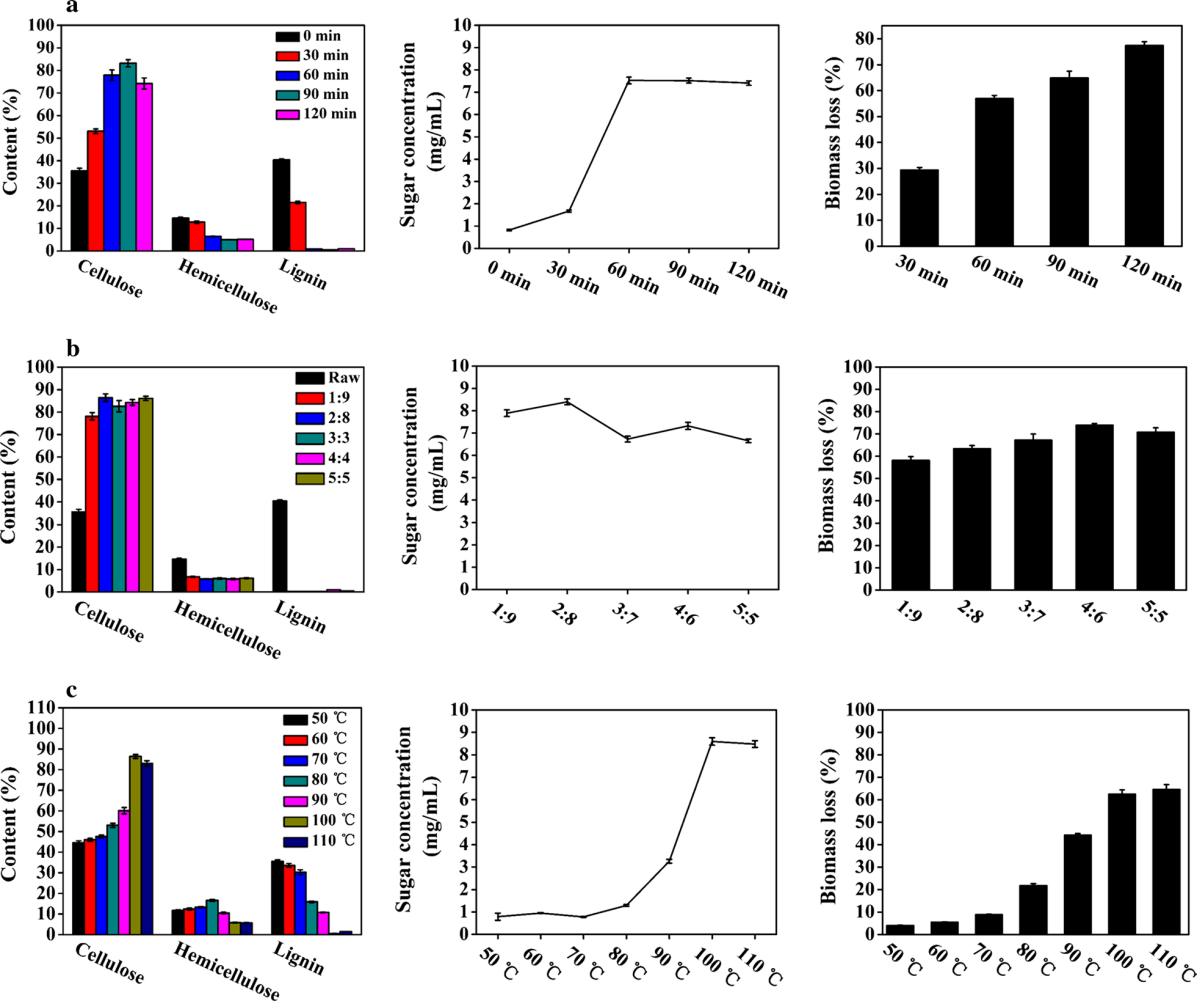
The changes of PH’s composition content, sugar concentration, and biomass loss at different pretreatment times (**a**), ratios of H_3_PO_4_ to H_2_O_2_ (v/v) (**b**), and temperatures (**c**)

To obtain higher yield of sugar, optimization of pretreatment conditions is necessary to remove lignin to the largest extent. In this work, three major factors, namely, pretreatment time, ratio of H_3_PO_4_ to H_2_O_2_ (v/v), and temperature were explored. Based on Table 1, pretreatment time was firstly optimized under the condition of 10% (w/v) of solid loading, ratio of H_3_PO_4_ to H_2_O_2_ (v/v, 1:9) at 100 °C. As is shown in Fig. 1a, the contents of cellulose (74.0–83.0%), hemicellulose (4.0–5.5%), acid-insoluble lignin (0.5–1.0%), and the concentration of sugar (7.4–7.6 mg/mL) were similar after 60, 90, and 120 min pretreatment, all of which was more effective than 30 min pretreatment. However, a lower biomass loss was observed for 60 min (56.9%) pretreatment compared with 90 min (64.9%) and 120 min (77.4%) pretreatments. Considering the overall results, 60 min was regarded as the most effective pretreatment time among 0–120 min.

**Table 1:** Pretreatment conditions at three kinds of factors for PH

Factors	Scope of optimizing factor	Immovable factors
Time	30, 60, 90, 120 min	10% (w/v) solid loading, ratio of H_3_PO_4_ to H_2_O_2_ (1:9), at 100 °C
Ratio of H_3_PO_4_ to H_2_O_2_ (v/v)	1:9, 2:8, 3:7, 4:6, 5:5	10% (w/v) solid loading, at 100 °C for 60 min
Temperature	50, 60, 70, 80, 90, 100, 110 °C	10% (w/v) solid loading, ratio of H_3_PO_4_ to H_2_O_2_ (2:8) for 60 min

In addition to pretreatment time, other two conditions, ratio of H_3_PO_4_ to H_2_O_2_ (v/v) and pretreatment temperature, were also optimized according to Table 1. As shown in Fig. 1b, c, in contrast to the ratio of H_3_PO_4_ to H_2_O_2_ (v/v), the temperature posed a great influence on the changes of components, biomass, and sugar concentration during the pretreatment process. In this study, 2:8 was verified as the optimal ratio of H_3_PO_4_ to H_2_O_2_ (v/v) due to the results that higher cellulose content (86.4%), lower hemicellulose (5.5%), lower acid-insoluble lignin content (0.2%), and higher concentration of sugar (8.4 mg/mL) were observed in comparison with other ratios. Three kinds of components and sugar yield were changed dramatically with different pretreatment temperatures, especially from 90–100 °C. At 100 °C, cellulose content occupied 86.4% (w/w) of the pretreated PH, which was approximately 1.4-fold higher than that of the raw PH. And sugar concentration generated from the pretreated PH achieved 8.6 mg/mL, which was about 10.5 times as much as the raw PH observed. Meanwhile, hemicellulose and lignin content decreased about 85.0 and 98.0%, respectively (Fig. 1c). However, these parameters remained steady as the temperature continued to rise. Thus, 100 °C was recognized as the optimal pretreatment temperature due to its lower energy cost and higher overall pretreatment efficiency.

In conclusion, the optimal pretreatment conditions were pretreatment time for 60 min, pretreatment temperature at 100 °C, 10% (w/v) of solid loading, and ratio of H_3_PO_4_ to H_2_O_2_ at 2:8 (v/v) corresponding to the concentrations (w/w) of H_3_PO_4_ and H_2_O_2_ with 23.10 and 21.85%, respectively.

### Comparisons with other pretreatment methods

**Fig. 2:**
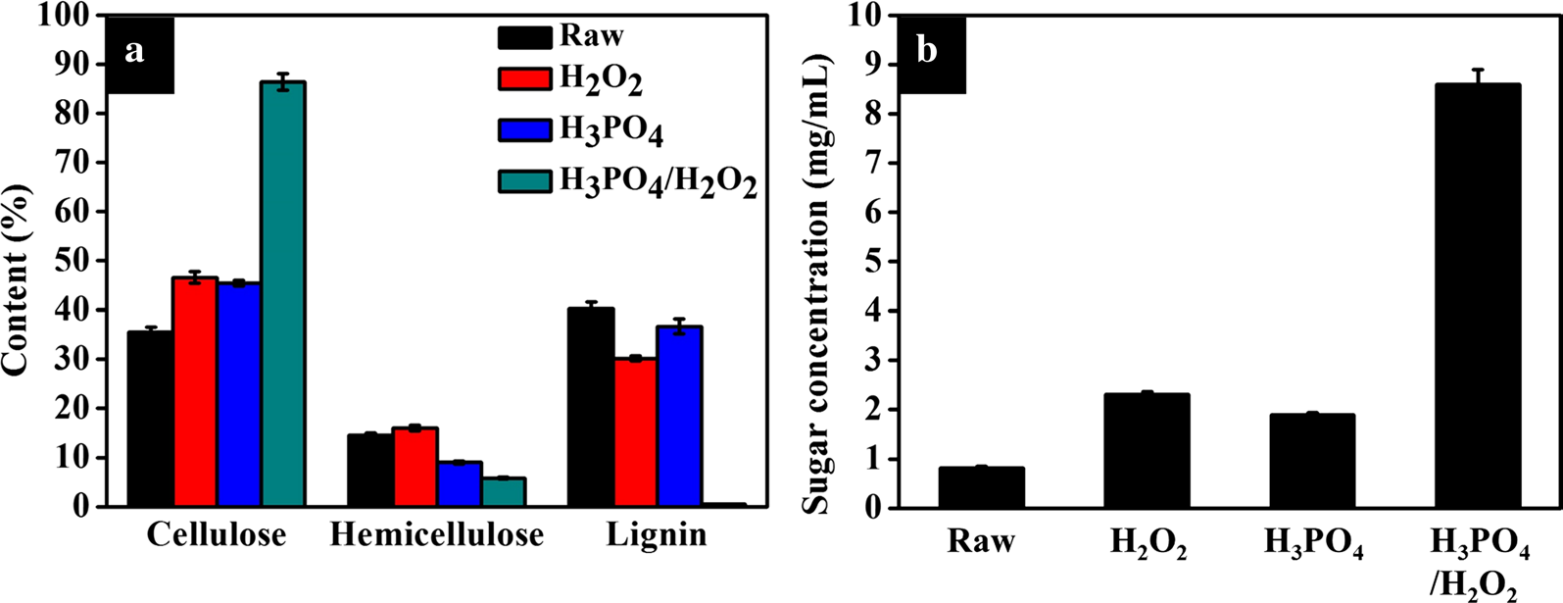
The changes of PH’s composition contents (**a**) and the concentration of sugar (**b**) at three kinds of pretreatment methods

Up to now, a huge number of pretreatment methods for lignocellulosic materials have been applied to improve the enzymatic hydrolysis efficiency with lower cost [26–31, 36]. Previous studies showed that dilute acid would be able to remove hemicellulose composition and H_2_O_2_ could reduce the lignin content under an appropriate condition [37, 38]. About 80–100% hemicellulose and 75% lignin of wheat straw were removed by the combination of concentrated H_3_PO_4_ (70–80%) and low concentration of H_2_O_2_ (1–5%) at about 40 °C for more than 2 h [39]. In this study, the PH biomass was pretreated with a mixture of lower concentration of H_3_PO_4_ (23.10%) and higher concentration of H_2_O_2_ (21.85%) at 100 °C for 60 min. The results demonstrated that more lignin (98.0%) was removed. Higher cellulose–glucose conversion (95.0%) was achieved with lower cellulase consumption (6.9 FPU/g glucan) using the pretreatment method in this research compared with the previous reports (about 77–94%) with 20 FPU/g glucan [39, 40]. Probably, the lignin removal could result in great increase of the enzymatic hydrolysis efficiency. Moreover, the changes of composition contents for PH pretreated by H_3_PO_4_ and H_2_O_2_ solely were also explored in this research. As shown in Fig. 2a, the cellulose content using separate pretreatment methods was a little higher than raw PH, but reached only about half of the combined pretreatment method. Besides that, separate pretreatment methods were less efficient in removing hemicellulose and lignin in comparison with the combined pretreatment method. As shown in Fig. 2b, higher sugar yield (8.6 mg/mL) was achieved when the PH was pretreated under condition of combined H_3_PO_4_ and H_2_O_2_ at the volume ratio of 2:8, in contrast to the H_3_PO_4_ (1.89 mg/mL) and H_2_O_2_ (2.31 mg/mL) pretreatment methods solely. The results demonstrated that the combination of H_3_PO_4_ and H_2_O_2_ pretreatment could remove the hemicellulose and lignin better and obtain much higher sugar yield than the separate pretreated PH.

Lignin, a complex polymer, is formed by oxidative coupling of three major C_6_–C_3_ units, namely, guaiacyl alcohol (G), syringyl alcohol (S), and* p*-coumaryl alcohol (H) [41]. The monolignols are linked by different ether bonds (such as aryl–aryl ether, alkyl–aryl ether, and biphenyl ether), C–C linkages (such as β–β and β-5), and the combination of ether bonds and C–C linkages (such as alkyl–aryl ether bond + β-5 carbon linkage and alkyl–alkyl ether bond + β–β carbon linkage). Among them, the aryl–aryl ether bond is the major interunit linkage [41, 42]. H_2_O_2_ could be decomposed into hydroperoxide anion (HOO^−^), hydroxyl radicals (HO·), and superoxide anion radicals (O_2_·^−^) by heating [41, 43]. Previous studies have reported that aryl ether bonds, lignin ring, ethylenic, carbonyl groups, and other linkages could be cleaved by HOO^−^ (strong nucleophile) and HO·, O_2_·^−^· (active radicals) generated from decomposed H_2_O_2_ [41, 44, 45]. Hence, part of lignin could be degraded with the treatment of H_2_O_2_. On the other hand, hemicelluloses were heterogeneous polysaccharides which contained either xylan or glucomannan backbones with acetyl group, galactose, arabinose, and methyl glucuronic acid on the side chains [46]. Different from the lignin and cellulose, hemicellulose could be ruined more easily by disrupting covalent bonds, hydrogen bonds, and van der Waals forces (such as C–O and C–H stretch in hemicellulose) during acidic pretreatment [46, 47]. Simultaneously, two main structures including aromatic ring and ring-conjugated C=C bonds of lignin were broken slightly, which resulted in a little lignin removal [47]. Moreover, the site of C_α_^+^ would be formed with the cleavage of α-aryl ether under acidic environment. The strong nucleophile, such as HOO^−^ from H_2_O_2_ decomposition, could attack the C_α_^+^ position to prevent the condensation with other lignin molecules again [48]. As indicated above, the condensed structure of lignocellulosic material was broken by removing hemicellulose using H_3_PO_4_, along with disrupting the lignin–carbohydrate linkages, and exposing Cα^+^ site, which might make it easier to remove the lignin using H_2_O_2_.

All the results indicated that, compared with mechanical pretreatment (milling and popping) and other chemical pretreatments (sulfuric acid, alkali, and ammonia fiber explosion), lower energy consumption, higher security, and greater efficiency for enzymatic hydrolysis were attained by the combination of H_3_PO_4_ and H_2_O_2_ pretreatment method.

### Surface morphology of raw and pretreated PH

**Fig. 3:**
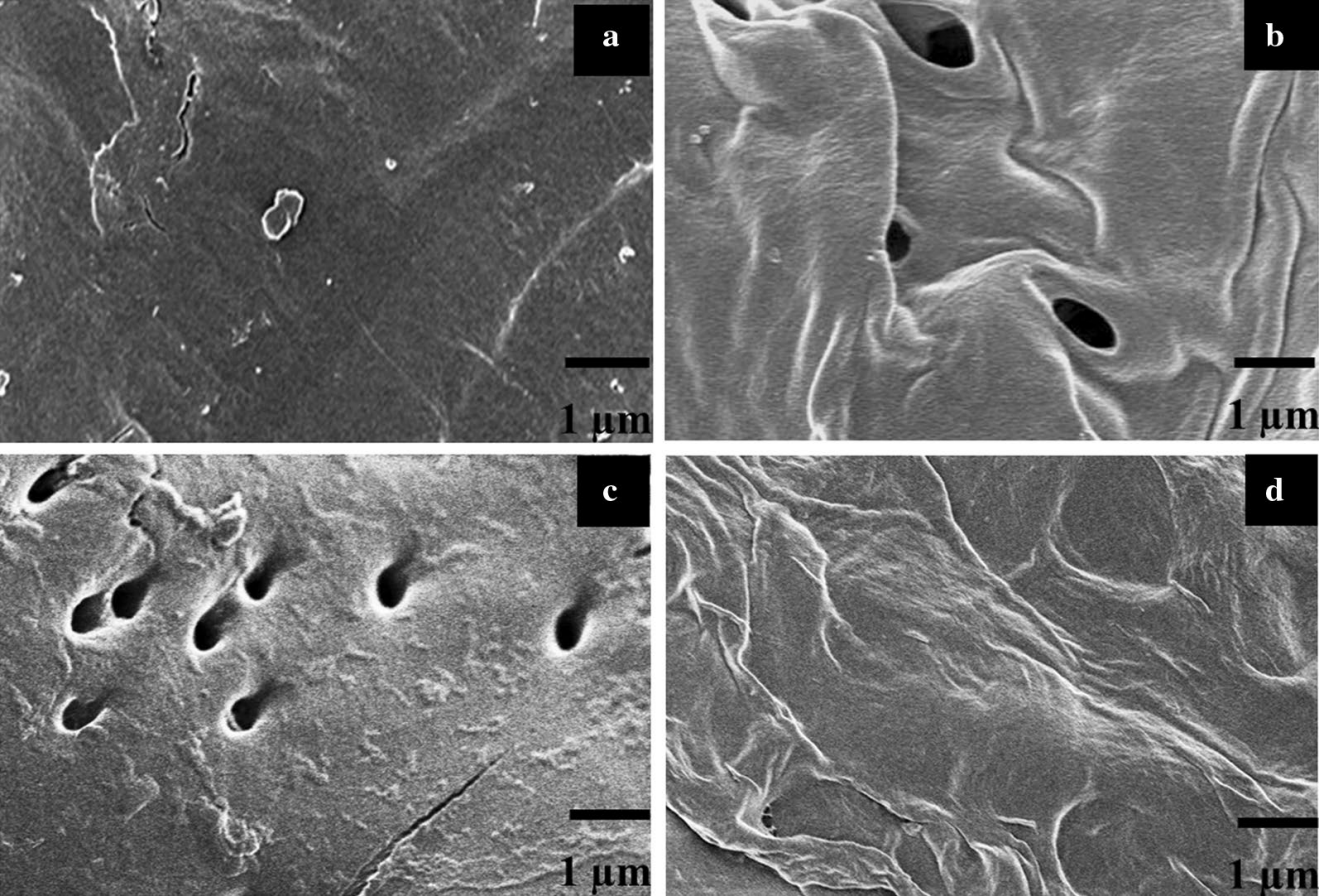
Scanning election microscopy (SEM) images of raw PH (**a**), H_3_PO_4_/H_2_O_2_-pretreated PH (**b**), popping-pretreated PH (**c**), and HPAC-pretreated PH (**d**). **c**, **d** were cited from our previous work [22], and were permitted to be used here

The enzymatic hydrolysis efficiency was determined by structure of the lignocellulosic materials [23]. The morphology changes of PH before and after being pretreated by H_3_PO_4_/H_2_O_2_ could provide direct information for explaining the rapid increase of the hydrolysis efficiency. Obviously, the surface of raw PH was smooth and integrated as shown in Fig. 3a. However, our previous study has proved that popping pretreatment led to the appearance of pores in the PH surface without thickness change (Fig. 3c). And the structure of the PH was still integrated though it became loosened and distortional after being pretreated by H_2_O_2_–acetic acid (HPAC) (Fig. 3d) [22]. In this study, significant pores occurred along with the pretreatment process by the combination of H_3_PO_4_ and H_2_O_2_ on account of hemicellulose removal. This pretreatment also resulted in the appearance of wrinkle on the surface of PH (Fig. 3a, b). We can safely come to the conclusion that structure of the PH was broken down and became much looser after being pretreated by H_3_PO_4_/H_2_O_2_. As a result, the enzymatic hydrolysis efficiency of the pretreated PH (95.0% glucose yield) increased much more than that of the raw PH (19.1% glucose yield) as hemicellulose and lignin were removed.

Solid-state cross-polarization magic angle spining carbon-13 nuclear magnetic resonance (CP/MAS ^13^C NMR) analysis of PH.

**Fig. 4:**
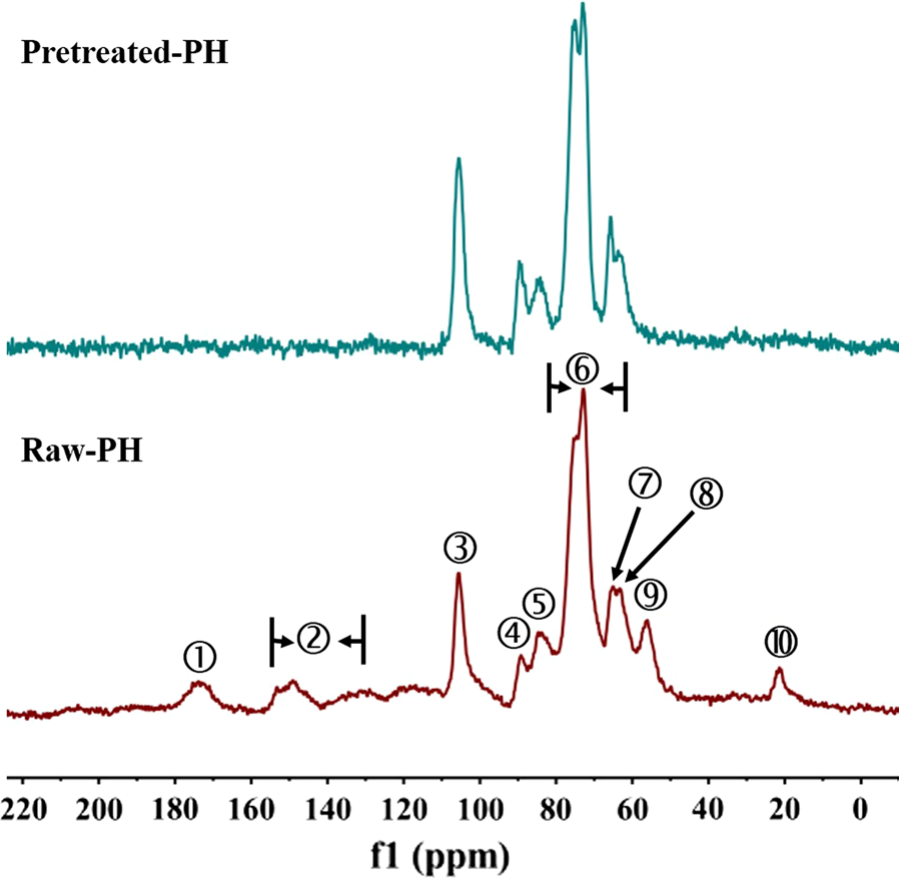
CP/MAS ^13^C NMR spectra of pretreated PH and raw PH

Solid-state NMR spectroscopy has been widely employed for structure characterization analysis of lignocellulosic materials during the past decades, and has always been considered a very useful analytical tool for carbohydrates and lignin [49]. Therefore, it was adopted to investigate the composition of cellulose, hemicellulose, and lignin before and after pretreating PH by the combination of H_3_PO_4_ and H_2_O_2_ in this work. As demonstrated in Fig. 4 and Table 2, various components were detected, whose chemical shifts were the same as the previous report [50, 51]. The two peaks at 173.8 and 21.42 ppm were assigned to hemicellulose. The peaks at 130–155 ppm, and 56.11 ppm were assigned to lignin. The peaks at 89.23 and 65.06 ppm represented crystalline cellulose, and the peaks at 84.00 and 63.20 ppm corresponded to amorphous cellulose. The peak at 105.53 ppm was from C1 of cellulose, while 72.89–75.38 ppm was from hemicellulose and cellulose. In comparison with the raw PH, the signal intensity of crystalline cellulose (89.23 and 65.06 ppm) increased while the amorphous cellulose (84.00 and 63.20 ppm) decreased. This might be attributed to the presence of water in the pretreatment procedure, leading to the thermodynamic instability of amorphous cellulose and its partial conversion into crystalline cellulose [52]. Furthermore, Wei et al. have reported that during the pretreatment process with H_3_PO_4_, the amorphous cellulose would be hydrolyzed when the temperature was above 50 °C [53]. Therefore, the cellulose crystallinity of pretreated PH increased with the pretreatment method conducted in this study. In addition, compared with raw PH, the signals of hemicellulose and lignin disappeared for pretreated PH, which indicated that the hemicellulose and lignin of PH have been degraded during the pretreatment process. Remarkably, the aromatic and methoxyl (OMe) groups of lignin at 130–155 and 56.11 ppm, respectively, were not detected in the pretreated PH either. All the results demonstrated that the complex network structure of the lignocellulose was destroyed through removing hemicellulose and lignin. Thus, it was deduced that the dramatic structure changes resulted from removal of hemicellulose and lignin might be the primary reason for the higher enzymatic hydrolysis efficiency and utilization of pretreated PH.

**Table 2:** Resonance assignments for the CP/MAS ^13^C solid-state NMR spectra of the pretreated and raw PH

No.	Chemical shift (ppm)	Functional group
➀	173.80	Acetate (C=O) of hemicellulose
➁	130–155	aromatic groups of lignin
➂	105.53	C_1_ cellulose
➃	89.23	C_4_ crystalline cellulose
➄	84.00	C_4_ amorphous cellulose
➅	72.89–75.38	C_2_, _5_, C_3_ of hemicellulose and cellulose
➆	65.06	C_6_ crystalline cellulose
➇	63.20	C_6_ amorphous cellulose
➈	56.11	OMe of lignin
➉	21.42	Acetate (CH_3_) of hemicellulose

### Cellulase adsorption of raw and pretreated PH

**Fig. 5:**
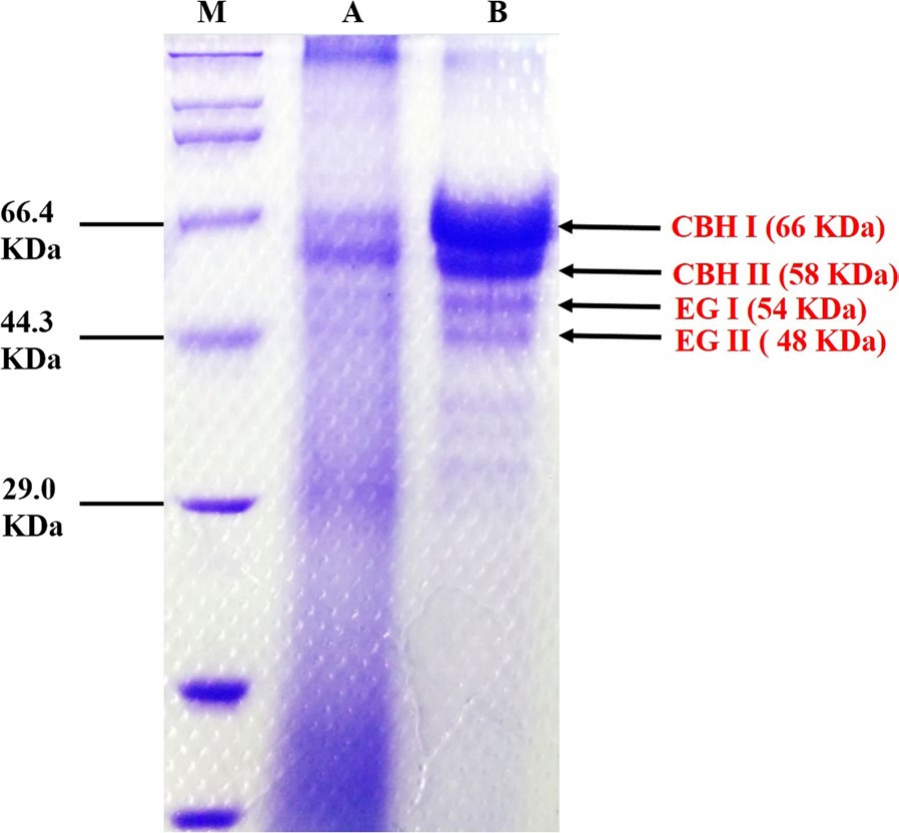
The comparison of cellulose adsorption before and after pretreating PH. Lane M, premixed protein marker (Broad); Lane A, cellulase adsorption of raw PH; Lane B, cellulase adsorption of pretreated PH

Sodium dodecyl sulfate polyacrylamide gel electrophoresis (SDS-PAGE) was conducted to detect the difference of cellulase adsorption before and after pretreating PH vividly. Previous studies reported that cellulase from *Trichoderma reesei* was a complex enzyme including at least five components (CBH I, CBH II, EG I, EG II, and EG III). Among them, CBH I, EG I, and EG II were regarded as the main components of cellulase [54–56]. In this research, four components of cellulase, CBH I, CBH II, EG I, and EG II, were detected to explore the cellulase adsorption of raw and pretreated PH. As exhibited in Fig. 5, the bands of two cellobiohydrolases (CBH I, 66 kDa and CBH II, 58 kDa) and two endoglucanases (EG I, 54 kDa and EG II, 48 KDa) in SDS-PAGE gel corresponding to the reported molecular mass were observed [54, 56, 57]. Moreover, the bands of four components in raw PH were significantly weaker than those in pretreated PH, showing that the adsorption of CBH I, CBH II, EG I, and EG II to pretreated PH was stronger than to raw PH. As reported, cellulose was surrounded by hemicellulose and lignin in lignocellulosic materials [23]. Thus, it was difficult for cellulose of raw PH to contact with cellulase efficiently. On the contrary, since nearly all the hemicellulose and lignin were removed during the pretreatment process, it was easier for cellulase to contact with and adsorb to the cellulose surface of pretreated PH.

### Inhibitor analysis

Various inhibitors would come up during the pretreatment process [58], especially when acetic acid, formic acid, levulinic acid, furfural, and 5-hydroxymethylfurfural (5-HMF) were generated during dilute acid pretreatment process at a high temperature [34]. Unfortunately, these inhibitors could restrict the growth and metabolism of *E. coli* by breaking down the single-strand DNA, inactivating the intracellular enzymes and reducing the intracellular pH [34, 58, 59]. In this study, after pretreated PH was hydrolyzed with cellulase, xylanase, and β-glucosidase, the enzymatic hydrolysate was analyzed using high-performance liquid chromatography (HPLC) to determine the categories and concentrations of inhibitors. Only three kinds of inhibitors, formic acid (5.4 × 10^−3^ mg/mL), levulinic acid (2.3 × 10^−2^ mg/mL), and furfural (2.8 × 10^−6^ mg/mL), were formed during the PH pretreatment procedure. Since the concentrations of inhibitors in the fermentation medium were much lower than the minimal inhibitory concentration (MIC) [60, 61], it was assumed that there might be no inhibition on *E. coli* fermentation to produce isoprene. Accordingly, lignocellulose pretreated by the combination of H_3_PO_4_ and H_2_O_2_ could be utilized to produce isoprene without removing the inhibitors with extra detoxification process.

### Isoprene production by different fermentation methods

**Fig. 6:**
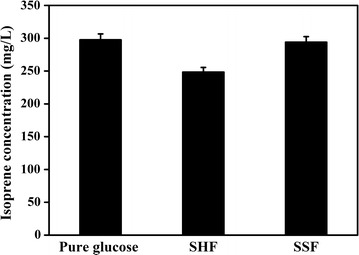
Isoprene production by three kinds of fermentation methods (pure glucose fermentation, SSF, and SHF). Cultures were induced at 30 °C for 24 h with 0.5 mM IPTG when OD_600_ reached 0.6. All the experiments were performed in triplicate

To test the efficiency of pretreated PH to produce isoprene, the engineered *E. coli* was utilized with separate hydrolysis and fermentation (SHF) and simultaneous saccharification and fermentation (SSF). In our previous study, the engineered strain, YJM25 (*E. coli* BL21™ (DE3)/pYJM21, pYJM14), was proved to be the optimal strain to produce isoprene, and the optimum glucose concentration in the fermentation medium was 3 g/L [21, 22]. Figure 6 shows that the isoprene yield by pure glucose fermentation was 298 ± 9 mg/L. 249 ± 6.7 and 294 ± 8.3 mg/L of isoprene were produced by the engineered strain with SHF and SSF methods, respectively, using the pretreated PH as the carbon source. The isoprene production via SHF and SSF had 8.3 and 9.8% glucose–isoprene conversions, equivalent to 83.5 and 98.8% of isoprene production via pure glucose fermentation, respectively. As the results showed, the glucose–isoprene conversion (9.8%) were higher than that (7%) published in the previous paper [21]. Obviously, SSF method had similar isoprene yield with pure glucose fermentation method, indicating that the inhibitors derived from pretreatment procedure had little effect on isoprene production. However, the isoprene yield of SHF method decreased compared with SSF method, which was consistent with the results shown in previous reports [62, 63]. All in all, the pretreatment strategy (H_3_PO_4_/H_2_O_2_) could be promisingly applied to pretreat the lignocellulose in isoprene production.

### Overall mass balance

**Fig. 7:**
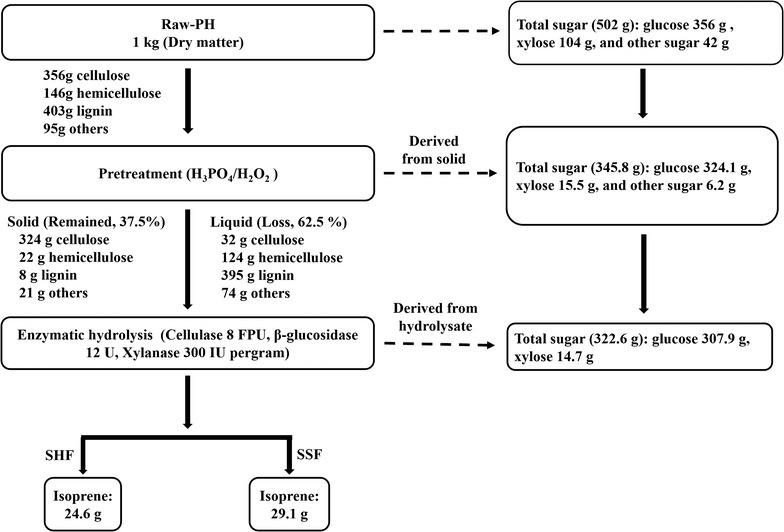
Overall mass balance of the PH by H_3_PO_4_ and H_2_O_2_ pretreatment

Ultimately, the overall mass balance for the operations including H_3_PO_4_/H_2_O_2_ pretreatment, enzymatic hydrolysis, and fermentation step was obtained. As indicated in Fig. 7, after being pretreated with H_3_PO_4_ and H_2_O_2_, the biomass loss of raw PH was about 62.5% containing about 39.5% lignin, 12.4% hemicellulose, 3.2% cellulose, and other 7.4% which were the organic solvent extractives (such as protein and fat) and ash. The remained solids of pretreated PH of 37.5%, including 32.4% cellulose, 2.2% hemicellulose, 0.8% lignin, and other 2.1% which were the organic solvent extractives (such as protein and fat) and ash, were collected. And then the remained cellulose was hydrolyzed into glucose for isoprene production. The glucose recovery was proximately 91.0%. Hence, hemicellulose and lignin were reduced by 85.0% (the remained hemicellulose 22 g, raw hemicellulose 146 g) and 98.0% (the remained lignin 8 g; raw lignin 403 g), respectively. The glucose content of pretreatment PH increased by about 2.4 times (86.4% of pretreated PH and 35.6% of raw PH) in contrast to that of the raw PH. Additionally, the glucose and xylose hydrolysis efficiency of pretreated PH were 95.0 and 94.8%, which increased 5 times and 11 times, respectively. On the other hand, compared with the isoprene yield fermented using pure glucose (298 ± 9 mg/L), 249 ± 6.7 and 294 ± 8.3 mg/L of isoprene were produced using the pretreated PH by SHF and SSF methods, respectively. The results demonstrated that the novel pretreatment method for lignocellulosic materials would have greater efficiency and advantages in isoprene production. Meanwhile, this strategy might also be widely used in other lignocellulosic materials to produce various biofuels.

In this study, high efficiency of isoprene production with PH using the newly developed pretreatment method was achieved. However, the mechanism of lignin removal for this method was unexplored because of the complexity of the lignin degradation products, which requires further study in the future.

## Conclusions

In this research, an optimized pretreatment method, the combination of H_3_PO_4_ and H_2_O_2_, was carried out to break down the concrete structure of lignocellulosic materials, and nearly all the hemicellulose and lignin of lignocellulose were removed. Meanwhile, the enzymatic hydrolysis efficiency of cellulose in pretreatment materials was increased dramatically compared with the raw materials. Intriguingly, only three kinds of inhibitors were generated during the pretreatment process, and the concentration of those inhibitors was too low to affect the isoprene yield. In addition, a higher isoprene yield was achieved with SSF method than that using SHF method. Conclusively, this novel pretreatment strategy could significantly contribute to the effective production of isoprene and other high-density biofuels in the future using lignocellulosic materials.

## Methods

### Materials

PH was milled to 40–60 mesh size and dried at 40 °C on oven before pretreatment. H_2_O_2_ and H_3_PO_4_ were purchased from Sinopharm Chemical Reagent Co., Ltd (Shanghai, China). All enzymes (cellulase, β-glucosidase, and xylanase) were obtained from Sigma (USA). The engineered strain YJM 25 (*E. coli* BL21™ (DE3)/pYJM21, pYJM14) was provided by Yang [21].

### H_2_O_2_/H_3_PO_4_ pretreatment

PH was pretreated using the pretreating solution (mixture of H_3_PO_4_ and H_2_O_2_). As shown below, three kinds of pretreating conditions were carried out step by step with 10% (w/v) solid loading: reaction times (30, 60, 90, and 120 min), ratios of H_3_PO_4_ to H_2_O_2_ (1:9, 2:8, 3:7, 4:6, and 5:5), and temperatures (10 °C gradient was set from 50–110 °C). After pretreatment, the remained PH was collected by filtration and dried completely at 40 °C for 24 h. The pretreated PH was stored at – 20 °C.

### Chemical composition analysis

Cellulose, hemicellulose, and lignin were analyzed with NREL Laboratory Analytical Procedure (LAP) [64]. 300 mg sample was mixed into 3 mL of 72% (w/w) sulfuric acid at 30 °C for 60 min. Then, the sulfuric acid was diluted to 4% by adding 84 mL deionized water. The mixture was incubated at 121 °C for 60 min. Then the mixture was cooled to room temperature. And the residue was removed by filtration and the supernatant was collected and determined by HPLC to measure the monomeric sugar content including glucose, xylose, arabinose, galactose, and mannose. The concentration of cellulose and hemicellulose was calculated according to the monomeric sugar content. Besides, the acid soluble lignin (ASL) content in the liquid was detected using UV–visible spectrophotometer. The residue was used to determine acid-insoluble lignin (AIL) content with muffle furnace at 575 ± 25 °C for 24 ± 6 h.

### Enzymatic hydrolysis

Cellulase from *Trichoderma reesei* ATCC 26921 (C8546, Sigma-Aldrich Corporation, St. Louis, MO, USA), β-glucosidase from *almonds* (49290, Sigma-Aldrich Corporation, St. Louis, MO, USA), and xylanase from *Trichoderma longibrachiatum* (X2629, Sigma-Aldrich) were used to hydrolyze raw and pretreated PH. Cellulase activity was represented with Filter paper unit (FPU/mL) and was measured using dinitrosalicylic acid reagent [65]. The activities of cellulase and β-glucosidase used in this study were 0.538 FPU/mg and 6 U/mg, respectively. Xylanase activity was defined as the amount of enzyme that produced 1 μmol of reducing sugar per 30 min, and Choi et al. have detected the activity of xylanase at 2.65 international units IU/mg [66, 67].

The PHs as 1% (w/v) substrate were treated in 50 mM sodium citrate buffer (pH 4.8) supplemented with 0.01% (w/v) sodium azide. The enzymes, cellulase, β-glucosidase, and xylanase, were loaded with 8 FPU/g, 12 U/g, and 300 IU/g of PH, respectively. All samples were completely suspended in rotary shaker at 200 rpm, 37 °C for 48 h. All enzymatic hydrolysis experiments were performed in triplicate. The concentration of reducing sugar was detected using dinitrosalicylic acid reagent [68]. The glucose concentration was determined using HPLC.

### Scanning election microscopy (SEM) analysis

The images of surface structure for raw and pretreated PH were obtained by scanning election microscopy (SEM; JSM-7500F, JEOL, Japan) at a beam voltage of 4 kV after the materials were dried completely at 50 °C for 24 h and coated by gold sputter (20 nm).

### Solid-state CP/MAS ^13^CNMR analysis

The powdered PH was analyzed with Bruker Avance III HD 500 MHz NMR Spectrometer at ambient temperature. CryoProbe™ Prodigy was used for ^13^C that employed both cross-polarization and magic angle spinning. 4 mm rotor was used in this detection. Acquisition time was 0.027 s, delay time was 5 s, and the proton 90° pulse time was 3.7 μs and 1024 scans for each sample. Mestrenova software was used to analyze the results.

### SDS-PAGE analysis of cellulase adsorption on PH

PH sample was incubated in 10 mL of 50 mM pre-chilled citrate buffer (PH 4.8) with 1% (w/v) loading at 4 °C, 150 rpm for 1 h. The solid PH was collected after centrifugation at 4 °C for 10 min, and then was washed three times with pre-chilled citrated buffer to get rid of the citrated buffer containing extra cellulase completely. Two kinds of PH were transferred into centrifuge tube including 400 μL of 2 × loading buffer (0.5 mL of 1 M Tris–HCl, 0.2 mg of SDS, 10 mg of bromophenol blue, 1 mL of glycerin, 0.1 mL of β-mercaptoethanol, and 3.4 mL deionized water), and then incubated in boiling water bath for 10 min. The supernatant was collected after centrifugation, and the proteins were separated using 4–12% SDS-PAGE and monitored with Coomassie brilliant blue R250 [17].

### Chromatography analysis

Glucose and inhibitors (formic acid, acetic acid, levulinic acid, furfural, and 5-HMF) were detected by (HPLC, Agilent 1200, USA) equipped with a refractive index (RID) detector. Bio-Rad Aminex HPX-87H column (7.8 mm × 300 mm, 9 µm) was used for glucose determination at 55 °C. A final 5 mmol/L of H_2_SO_4_ was used as the mobile phase at a flow rate of 0.5 mL/min. Formic acid, acetic acid, and levulinic acid were determined with AS11HC column which eluted with 80% (v/v) water and 20% (v/v) of a mixture containing 0.4 mM NaOH and methanol (50% v/v) at a flow rate of 1.4 mL/min.

The concentrations of furfural and 5-HMF were determined with C-18 column (Nucleosil 100-5 C18, Merck, Darmstadt, Germany) with a gradient of 5–100% (v/v) methanol and 0.025% (v/v) of trifluoroacetic acid at a flow rate of 0.8 mL/min. Glucose and inhibitors were identified according to the retention time of standard samples and the concentration was calculated with peak area based on a standard curve.

Gas sample (1 mL) was analyzed using headspace sampling by Gas chromatograph (GC7900, Shanghai, China). Isoprene was separated using TM-WAX column (25 m × 0.25 mm × 0.25 μL). Flame ionization detector (FID) was used to detect the isoprene and N_2_ was used as a carrier gas. The column temperature was initially set at 50 °C for 1 min and was increased to 80 °C at a rate of 6 °C/min. The injector temperature was 140 °C and the detector temperature was 230 °C. The isoprene concentration was calculated by converting GC peak area to milligram of isoprene via a calibration curve.

### Fermentation

SSF and SHF were carried out using a series of 25 mL sealed shake flasks containing 5 mL fermentation medium which included enzymatic hydrolysate or dry pretreatment materials with 3 g/L of the glucose, cellulase (8 FPU/g), β-glucosidase (12 U/g), xylanase (300 IU/g), K_2_HPO_4_ 9.8 g/L, beef extract 9 g/L, ferric ammonium citrate 0.3 g/L, citric acid monohydrate 2.1 g/L, MgSO_4_ 0.06 g/L, and 1 mL trace element solution consisting of (NH_4_)_6_Mo_7_O_24_·4H_2_O 0.37 g/L, ZnSO_4_·7H_2_O 0.29 g/L, H_3_BO_4_ 2.47 g/L, CuSO_4_·5H_2_O 0.25 g/L, and MnCl_2_·4H_2_O 1.58 g/L. Meanwhile, the medium contained 34 mg/mL chloramphenicol and 100 mg/mL ampicillin. The engineered *E. coli* strain YJM25 was inoculated in the broth culture and placed in a gyratory shaker incubator at 37 °C and 180 rpm. When OD_600_ reached 0.6, IPTG was added in the final concentration of 0.5 mM, and the culture was further incubated at 30 °C for 24 h. All experiments were performed in triplicate.

## Abbreviations

PH: peanut hull
SHF: separate hydrolysis and fermentation
SSF: simultaneous saccharification and fermentation
H_3_PO_4_: phosphoric acid
H_2_O_2_: hydrogen peroxide
HPAC: H_2_O_2_–acetic acid
SEM: scanning election microscopy
CP/MAS^13^CNMR: solid-state cross-polarization magic angle spinning carbon-13 nuclear magnetic resonance
OMe: methoxyl
SDS-PAGE: sodium dodecyl sulfate polyacrylamide gel electrophoresis
5-HMF: 5-hydroxymethylfurfural
HPLC: high-performance liquid chromatography
MIC: minimal inhibitory concentration
ASL: acid soluble lignin
AIL: acid-insoluble lignin
FPU: filter paper unit
FID: flame ionization detector
GC: gas chromatograph
G: guaiacyl alcohol
S: syringyl alcohol
H: *p*-coumaryl alcohol
HOO^−^: hydroperoxide anion
HO^•^: hydroxyl radicals
O_2_^−•^: superoxide anion radicals
